# Effects, Adherence, and Therapists’ Perceptions of Web- and Mobile-Supported Group Therapy for Depression: Mixed-Methods Study

**DOI:** 10.2196/11860

**Published:** 2019-05-06

**Authors:** Raphael Schuster, Inanna Kalthoff, Alexandra Walther, Lena Köhldorfer, Edith Partinger, Thomas Berger, Anton-Rupert Laireiter

**Affiliations:** 1 Center for Clinical Psychology, Psychotherapy and Health Psychology Department of Psychology University of Salzburg Salzburg Austria; 2 Department of Applied Psychology: Health, Development and Promotion Faculty of Psychology University of Vienna Vienna Austria; 3 Department of Clinical Psychology and Psychotherapy University of Berne Berne Switzerland

**Keywords:** eHealth, mobile phone, computer-assisted therapy, monitoring, group therapy, depression, professional-patient relations

## Abstract

**Background:**

Blended group therapy (bGT) has been investigated a several times for anxiety and depression, but information on patients’ adherence to and therapists’ perception of the novel format is nonexistent. Furthermore, many studies investigated mainly female and highly educated populations, limiting the validity of previous findings.

**Objective:**

This study aimed to reduce the gaps and limitations of the previous findings by evaluating an integrated internet- and mobile-supported bGT format.

**Methods:**

A total of 27 patients diagnosed with major depression (14/27, 52% female and 7/27, 25.9% compulsory education) participated in a 7-week treatment at a university outpatient clinic. Furthermore, 8 novice therapists participated in semistructured interviews and a subsequent cross-validation survey.

**Results:**

Primary symptom reduction was high (*d*=1.31 to 1.51) and remained stable for the follow-up period. Therapists identified advantages (eg, patient engagement, treatment intensification, and improved therapeutic relation) and disadvantages (eg, increased workload, data issues, and undesired effects) of bGT. The required online guidance time was 10.3 min per patient and week, including guidance on exercises (67% or 6.9 min) and intimate communication (33% or 3.4 min). Concerning patients’ adherence to bGT, tracked completion of all Web-based and mobile tasks was high and comparable with group attendance.

**Conclusions:**

Results suggest high feasibility of bGT in a gender-balanced, moderately educated sample. bGT provides group therapists with tools for individual care, resulting in an optimization of the therapy process, and high completion rates of the implemented bGT elements. The limited work experience of the involved therapists restricts the study findings, and potential drawbacks need to be regarded in the development of future bGT interventions.

## Introduction

### Background

Depression is one of the most prevalent mental disorders and a leading cause of disability. It imposes suffering and high costs on individuals, societies, and health systems [1]. In line with international research priorities [2,3], different forms of mobile- and internet-based interventions constitute innovative and efficient strategies to deliver evidence-based psychological treatments for common mental health disorders [4-8].

Among their most frequent formats, mobile and Web-based interventions offer flexible and anonymous access to mental health services, resulting in low social barriers and low risk of stigmatization [9,10]. Owing to the high degree of automatization, those interventions guarantee standardized treatments to a highly scalable extent. These properties make them attractive for mental health care organizations and have led to the launch of the first routine online clinics [11-13].

However, Web-based and mobile interventions also exhibit limitations as they do not meet all patients’ needs and preferences, and therapist contact usually is restricted to a wide degree. Furthermore, therapeutic guidance frequently is associated with better treatment outcomes and reduced dropout rates [14,15]. Finally, many therapists lack experience with this novel approach and hold more cautious attitudes toward Web-based interventions [16]. Comparable levels of caution and awareness have been found among different interest parties (eg, mental health care providers and policy makers), contributing to a frequently discussed retardation of dissemination efforts [17]. Therefore, it is crucial to gain further insight into therapists’ perception and acceptance of technology-aided treatments.

**Figure 1:**
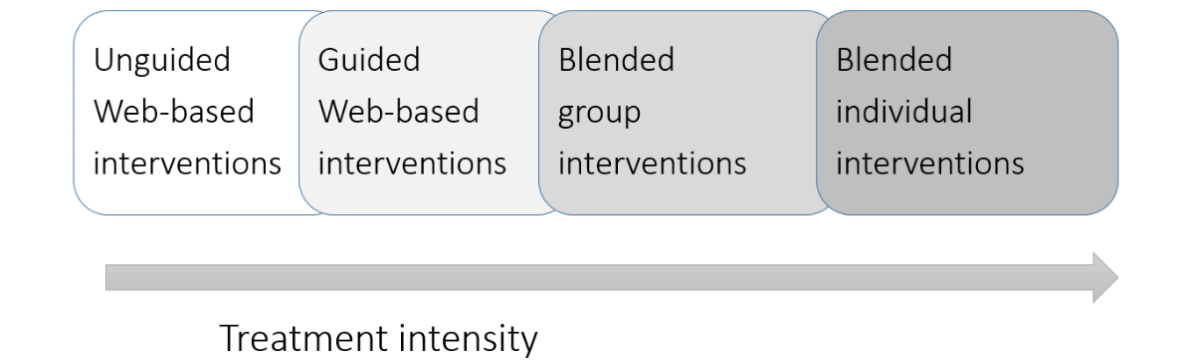
Blends of Web-based and face-to-face therapy.

The techniques developed in the field of Web-based therapy [7,18-21] can also be harnessed to improve existing forms of face-to-face therapy, resulting in a continuum of blended treatments (Figure 1). Within blended interventions (synonymous computer- or mobile-supported interventions), the spectrum of possible applications ranges from adjuncts to psychotherapy [22], which can be applied before, after, or during treatment [23,24], to more integrated forms of therapy in which Web-based or mobile elements and personal sessions are more deeply intertwined into one treatment rationale [25-27]. A growing number of studies show that blended interventions can lead to shortened treatments in which less therapist time is needed to achieve substantial effects [23,26,28]. Simultaneously, Web-based or mobile elements can be deployed to optimize the therapeutic process, to foster transfer, and to boost effects of classical treatments. In this regard, first studies in routine care found additive effects of traditional face-to-face therapy augmented with Web-based therapy elements [29,30].

According to therapists, patients can profit from blended interventions in the form of increased treatment accessibility and flexibility, as well as from the improvement of patients’ self-management and the optimal use of face-to-face sessions [31,32]. Additionally, mental health care providers, policy makers, and other such organizations seem to have a more positive conception of blended therapy compared with pure Web-based therapy [17], and therapists seem to prefer the blended format because it is associated with less risks (eg, diagnostic process) [16]. Among the potential disadvantages of the integrated format, therapists frequently remark that blended therapy is not feasible for all patients and that the format at times could hamper the therapeutic process—in particular, the establishment of the therapeutic alliance [31,32]. Consequently, those issues should be investigated in more detail in patient- and therapist-related studies.

While most blended research focuses on individual therapy [23], less is known about its potential for group therapy. Psychological groups have a broad range of applications in inpatient and outpatient settings [33]; and the spectrum ranges from informational groups, over psychoeducational groups, to group counseling and group psychotherapy [34]. So far, the feasibility and effects of blended group therapy (bGT; synonymous computer- or app-supported group therapy) have been investigated in terms of brief interventions for depression and anxiety. For example, computer-based relaxation, cognitive restructuring, and self-control desensitization have been found to be supportive in the treatment of generalized anxiety disorder (GAD) [35]. Furthermore, first evidence for the efficiency of brief bGT for social anxiety disorder and GAD was found in small comparative trials, leading to significant symptom reductions in a comparably short time period [36,37]. As for depression, several feasibility studies investigated the merits of supportive computer- and mobile-based elements. For example, a tablet-guided behavioral activation (BA) intervention was found to be feasible for the treatment of major depressive disorder [38]. In another study, Aguilera et al found beneficial effects of group therapy augmented with monitoring and text messaging [39]. Furthermore, a brief resource-oriented bGT intervention was developed by our workgroup to address depression by means of a low-threshold, stigma-free treatment strategy. The results revealed high feasibility in terms of client satisfaction and observed between-group treatment effects. Moreover, assessed parameters of treatment adherence (eg, self-reported exercising) indicated high acceptability of bGT elements [40-41]. In a subsequent qualitative investigation (including 13 patients of this study), the use of technology was described as a therapeutic factor, facilitating insight, exercising, and treatment transfer [42].

### Objectives

This study wants to carry this work forward by investigating an integrated bGT intervention based on Acceptance and Commitment Therapy (ACT) principles [43], complemented with elements of BA [44]. As in previous studies, patients’ self-reported depressiveness and general health, as well as ACT-specific variables and standardized measures for service satisfaction and usability, were assessed. For the first time, log data were tracked to provide reliable information on completion rates of computer- and mobile-based elements. Focusing on the therapist-related feasibility of bGT, this study includes therapist interviews and a subsequent follow-up survey. As a related aspect, the amount of weekly online guidance was recorded to ascertain therapists’ between-session workload.

## Methods

### Participants

The trial was preregistered at the German trial register (DRKS Number: DRKS00010888), and the regional ethics committee of the University of Salzburg approved the study procedure. Participants were recruited via a multimodal recruitment strategy by handing out flyers in public health centers and densely populated public areas and by advertisements on depression-related Web pages. After registering on the study platform participants obtained detailed information about the procedure and goals of the study and were asked to give informed consent.

The selection of participants followed 2 steps. Participants were asked to fill out a short screening questionnaire. This included the short version of the Center of Epidemiologic Studies Depression (CES-D) scale [45,46] and additional questions regarding current and past psychological or medical treatment. Participants reporting at least mild levels of depression (CES-D>17) and no suicidal ideation, critical drinking, or past or recent history of severe psychiatric conditions were invited to take part in a diagnostic interview.

**Figure 2:**
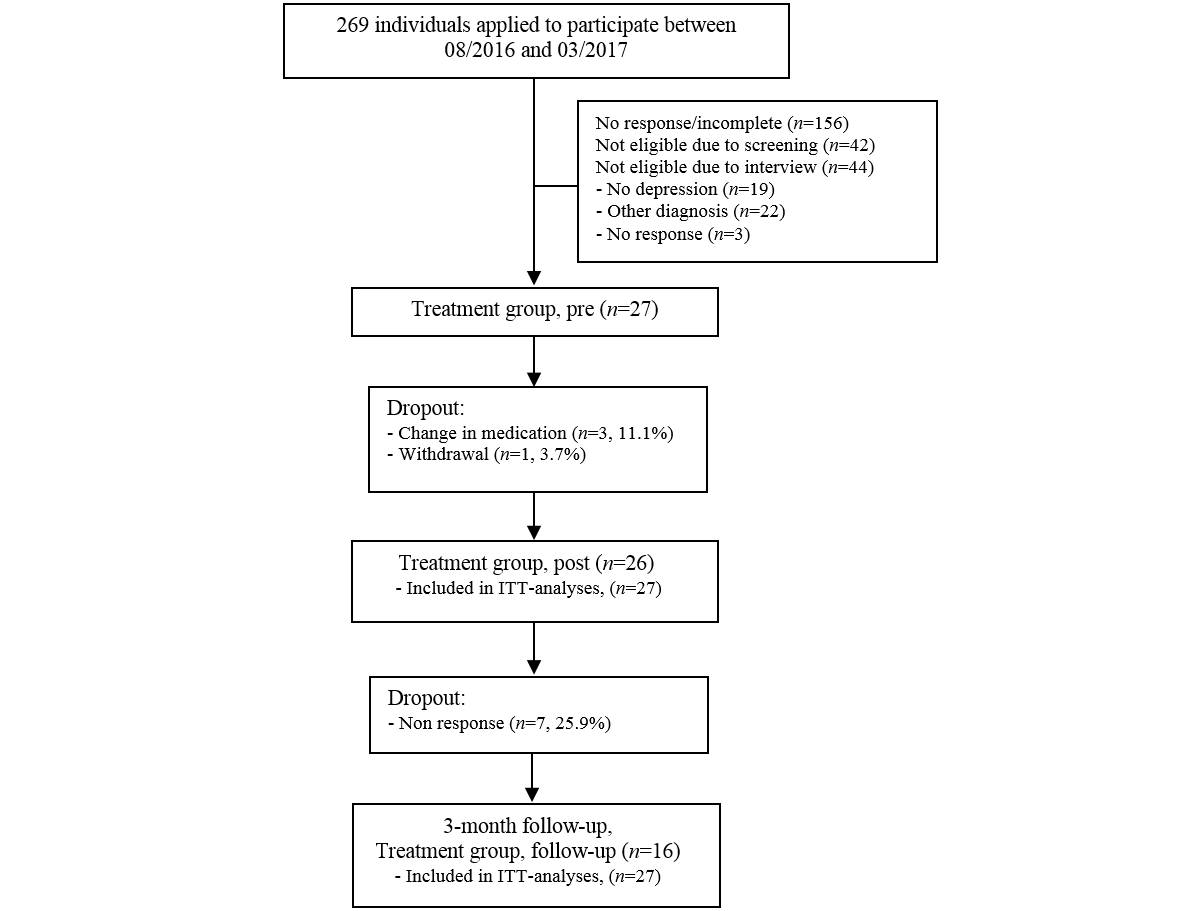
Study flow chart. ITT: intention to treat.

Personal clinical interviews were conducted by 3 independent and experienced psychologists, applying the German Mini-Diagnostic Interview for Psychological Disorders (DIPS) [47]. The Mini-DIPS is a 30- to 45-min version of the German DIPS [48], based on the International Classification of Diseases-10 depression criteria. Participants were deemed eligible if the following criteria applied: aged between 18 and 65 years, suffering from mild-to-moderate levels of major depression and/or dysthymia; and/or mild-to-moderate comorbid anxiety, as well as familiarity with the use of personal computers and possession of a smartphone. According to clinical judgement, participants were excluded if they suffered from severe depression (>7 criteria, including main symptoms), severe anxiety disorder, bipolar disorder, any schizoaffective disorder, severe psychiatric and psychotic conditions, substance abuse, suicidal ideation, or if they exhibited low German language and/or computer skills. Participants were also excluded if they currently underwent psychotherapy. Psychiatric medication was tolerated but has been kept constant for at least 3 months before study onset. Figure 2 presents the flowchart demonstrating the recruitment and research procedure in detail.

### Procedure

After preassessment, participants were provided with access to the internet platform (Minddistrict) and scheduled to one of 2 weekly groups, depending on personal preferences. To provide personal support in case of technical problems, the app-based diary was installed at the end of the first group session. Group meetings lasted 7 weeks, and each session was preceded by a preparatory Web-based module. The therapist gave supportive feedback after completion of a given Web-based session and occasionally gave reminders to participants by sending out prompts via the platform. The app-based diary complemented the blended treatment with a focus on the transfer of previously learned techniques into daily life. Participants were free to logon to the platform after treatment had ended but did no longer receive therapist guidance. As recommended by several guidelines [33], group sessions were held in a double trainer format which lasted 90 min each. One week after the last group session, the Web-based post assessment had to be filled out and follow-up assessment took place 3 months later.

### Intervention

**Figure 3:**
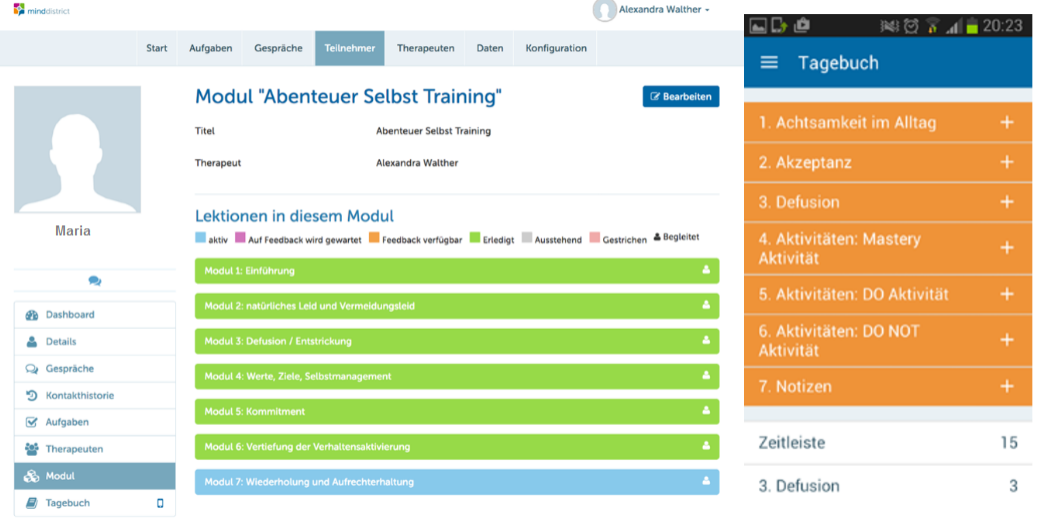
User interfaces of the web-based platform and the smartphone app.

The 7 weeks intense group treatment was based on the ACT and BA principles. ACT [43] is one of the several new treatments originating from cognitive behavioral therapy (CBT). The core principles of this contemporary approach can be divided into mindfulness and acceptance techniques (acceptance, cognitive defusion, and self as context) and behavior change techniques (contact with the present moment, values, and committed action). Even though ACT and BA diverge regarding certain theoretical assumptions (ie, proposed mechanisms of action) [49], they also share many communalities (eg, clarification of goals or strong emphasis on behavioral techniques). Therefore, ACT-based behavior change techniques can be complemented by BA principles [50]. The current treatment rationale was recreated based on a previous intervention, merging ACT and BA into one integrated rationale [51]. Detailed information on intervention content and design can be obtained from Table 1 and Figure 3.

With regard to the use of computer- and mobile-based elements, the patients’ weekly routine consisted of 3 steps. First, a preparatory Web-based module, featuring video clips, text-based tasks, and an asynchronous therapist chat, had to be completed. Afterward, patients received individualized feedback from the assigned therapist (if applicable within 2 days). Second, patients participated in the weekly reunions, which again were partially complemented by modern media (ie, short clips or PowerPoint presentations). As a last step, patients were guided by weekly mobile phone diary tasks, which were scheduled for 7 days following the weekly group session. All reminders and prompts were modifiable according to personal preferences, and wherever possible, therapists were instructed to balance media and personal treatment elements according to patient needs and their professional judgement. If patients did not adhere to the Web-based tasks, therapists were instructed to send out a prompt in the middle of the treatment week and, again, once on the day before the forthcoming group session. If patients complained about the number of reminders, the prompts were reduced or stopped.

**Table 1:** Group sessions and computer and multimedia elements of the intervention.

Week	Web-based module	Group session	App	Workbook
1	Introduction into mindfulness	Introduction into ACT^a^, mindfulness	Feature 1: Mindfulness in daily life	List of mindful activities
2	Natural suffering and suffering through avoidance	Avoidance and acceptance	Feature 2: Acceptance	Acceptance of a difficult situation, topic, character trait, or conflict; Reflection on mindfulness
3	Defusion	Fusion and defusion	Feature 3: Defusion	Typical examples of defusion
4	Values, goals, and self-management	Values, mastery, and self-management	Feature 4: Mastery activities	Bull’s-eye exercise; Example and sheet for SMART^b^ principle; Activity planning
5	Commitment	Commitment and positive reinforcement	Feature 5a: “Do activities” Feature 5b: “Do not activities”	Determination, ranking, and planning of do- and do not activities; Self-management; Activity planning
6	Expansion of behavioral activation	Expansion of behavioral activation	Continuation of previous features of the app	Contracts
7	Review and transfer	Transfer and conclusion	Continuation of previous features of the app	Plan for relapse

^a^ACT: Acceptance and Commitment Therapy.

^b^SMART: frequent self-management principle.

### Therapists

A total of 8 novice therapists (2 male and 6 female) conducted the groups in a double trainer setting. Of the 8 therapists, 2 finished their master’s degree (MSc) or Doctor of Science in psychology and underwent tertiary training in psychotherapy (CBT), clinical psychology (CBT), or medicine at the time of the intervention. The remaining 5 therapists were in their final year of clinical psychology (MSc) and had clinical experience with conducting classical forms of individual or group therapy, as well as with drafting psychological expert reports. None of the therapists had previous experience with conducting bGT or any other form of Web-based therapy. All participating therapists underwent previous training (minimum 40 hours), including a 6-digital versatile disc ACT-series (ACT in Action) and 2 textbooks [52,53] encompassing sections on difficult situations in the ACT. Therapists also protocolled their weekly group sessions. Adherence to the foreseen treatment course was supported by in- and between-session media and technology elements. Of the 8 therapists, 2 therapists participated in a previous bGT study [41] and 6 participated in this study. At the time of the therapist interviews (3 to 12 months after study end), all except 1 therapist were in tertiary clinical trainings for psychotherapy (3 therapists; CBT and client-centered therapy), clinical psychology (3 therapists; CBT), or medicine (1 therapist).

### Outcome Measures

#### Primary Outcomes

The principal outcome of the study was reduction of depressed mood. It was measured by the short version of the German translation of the CES-D scale [45,46]. This questionnaire measures interactive, cognitive, and somatic symptoms, as well as emotions and motor functions related to depression. The 16 items are rated on a 4-point Likert scale. Any value above 17 is interpreted as critical. The German version’s critical threshold (>17) has high discriminative validity, pointed out by a sensitivity of 90%, a specificity of 87% [45], and an area-under-the-curve value of 0.94 [54]. The reliability of the CES-D has been shown to be high [46]. The Cronbach alpha in this study was .90.

As a more general self-report questionnaire that measures psychological distress, nonspecific current mental health, and the risk of developing psychological disorders, the General Health Questionnaire-12 (GHQ-12 [55]) was used. The questionnaire has shown solid reliability [56] and good intercultural validity [57]. The Cronbach alpha in this study was .84.

#### Secondary Outcomes

Psychological flexibility (ie, acceptance of unpleasant feelings, worry, and control agendas) is the central psychological construct of the ACT and was measured by the Fragebogen zu Akzeptanz und Handeln II [58]. This is the German version of the Acceptance and Action Questionnaire-II (AAQ-II) by Bond et al [59]. The 7 items are rated on a 7-point Likert scale. The Cronbach alpha of the this study was .89.

Anxiety was measured with the Anxious Thoughts Inventory (AnTi) [60] (German translation [61]). It analyzes 3 dimensions of worry: social worry, physical health worry, and meta-worry (worry about worries). The 22 items are rated on a 4-point Likert scale. The Cronbach alpha in this study was .87.

Finally, worry was measured with the PSWQ-3 [62], a short form of the Penn State Worry Questionnaire [63]. It is a questionnaire that assesses self-reported key aspects of worry in GAD. The 3 items are rated on a 5-point Likert scale. The Cronbach alpha in this study was .74.

### Client Satisfaction and System Usability

System usability of applied app and Web elements was measured by the System Usability Scale (SUS) [64]. The SUS is a robust questionnaire with 10 items rated on a 5-point Likert scale. The sum score ranges from 1 to 100. SUS scores >85.5 classify excellent usability; scores ≤85.5 and >71.4 classify as good, scores ≤71.4 and >50.9 as OK, scores ≤50.9 and >35.7 as poor, and scores ≤35.7 and >20.3 as awful [65]. The Cronbach alpha in this study was .78.

The ZUF-8 (Fragebogen zur Patientenzufriedenheit) [66], the German version of the Client Satisfaction Questionnaire-8 [67], was used to assess several aspects of participants’ overall treatment satisfaction. The 8 items are rated on a 4-point Likert scale. The total score can range from 8 to 32, with a cut-off value of 24 [68] to grade a person as dissatisfied. The Cronbach alpha in this study was .94. For the mobile-based app, adherence was defined as more than 3 weekly entries.

### Statistical Analyses

SPSS 24 (SPSS Inc) was used to carry out the analyses. Significant differences between pre, post, and follow-up were analyzed by linear mixed models, with compound symmetry as covariance type and restricted maximum likelihood estimation. Missing outcome values were analyzed according to the intention-to-treat (ITT) principle. Individual pre to post changes served as a base for the reliable change indexes (RCIs) [69]. We used internal consistency as a parameter for RCI reliability [70]. The reliable change criteria were 5.87 scale points for the CES-D and 4.87 for the GHQ-12. For the assessment of change, within-group effect sizes were calculated with pooled SD and reported in Cohen *d* [71]. Power analysis was executed with G*Power [72]. We assumed that the effect size for the secondary outcomes may only lie in the medium range. Thus, an estimated sample size of *N*=22 was calculated for a medium within-subjects effect size of *d*=0.65 (alpha error=.05; power *beta*=.90).

### Qualitative Analyses

On the basis of a structured interview guide (Multimedia Appendix 1), audiotaped therapist interviews were conducted by the first author (RS). Interviews lasted between 28 and 56 min (*mean* 44) and were transcribed by 2 independent psychologists who also analyzed the material obtained. MAXQDA was used to conduct the analysis. Analysts were blind to the outcomes and identity of participants. The qualitative content analysis [73] served as the method of information extraction by applying a deductive extraction based on the addressed research questions. After analyzing one third of the transcript, both psychologists and the first author (RS) jointly revised the code system to reach agreement on the applied coding system. Principal codes closely related to the structured interview guide were then specified into further emerging subthemes. After content analysis, a set of follow-up questions was surveyed anonymously to depict the degree of consensus on particular findings among the interviewed therapists. Of the 30 items, 10 items related to design aspects will be reported in a further publication on bGT design. The complete list of follow-up questions was translated by a bilingual psychologist and is presented in (Multimedia Appendix 2)*.*

## Results

### Participants

A comprehensive overview of participant characteristics at baseline is provided in Table 2. Men and women were equally represented (52%, 14/27 female,), with a mean age of 37.7 years (*SD* 13.7), and relatively low levels of education and employment status. Furthermore, 1 patient withdrew from treatment, resulting in a completion rate of 96% (26/27). During the study period, 3 patients reported changes in medication. According to ITT principles, those patients remained in the analyses. Detailed information on participants’ enrolment and participation throughout the study can be seen in Figure 2.

**Table 2:** Demographic, behavioral, and clinical characteristics of the sample at pretreatment (N=27).

Characteristic		Statistics
Age (years), mean (SD)		37.70 (13.66)
Gender, female n (%)		14 (51.9)
**Education, n (%)**		
	≥9 years (compulsory school)	7 (25.9)
	≥12 years (A level)	12 (44.4)
	≥any tertiary education (eg, university)	8 (29.6)
**Employment, n (%)**		
	Full time	11 (40.7)
	Part time	6 (22.2)
	None/marginally	5 (18.5)
	Currently in education	5 (18.5)
Current psychopharmacological treatment, n (%)		3 (12)
Previous psychotherapeutic treatment, n (%)		14 (54)
**Computer experience, n (%)**		
	Daily use	25 (92.6)
	Weekly use	2 (7.4)
**Diagnosis, n (%)**		
	F32.0 (mild depressive episode), n (%)	3 (11.1)
	F32.1 (moderate depressive episode), n (%)	8 (29.6)
	F33.0 (recurrent depressive episode, current episode mild), n (%)	10 (37.0)
	F33.1 (recurrent depressive episode, current episode moderate), n (%)	4 (14.8)
	F33.4 (recurrent depressive disorder, in remission—elevated levels of depression), n (%)	2 (7.4)
**Comorbidities, n (%)**		
	F10.1/2 (harmful use of alcohol/addiction)	1 (3.7)
	F40.0 (agoraphobia without panic disorder)	1 (3.7)
	F40.1 (social phobia)	2 (7.4)
	F40.2 (specific phobia)	1 (3.7)
	F41.1 (generalized anxiety disorder)	3 (11.1)
	F43.2 (adjustment disorder)	1 (3.7)
	F50.2 (bulimia nervosa)	1 (3.7)

### Primary and Secondary Outcomes

Linear mixed models unveiled significant changes in all outcome measures, and pre- to posteffect sizes for primary outcomes were large to very large (*d*=1.31 to 1.51). The primary outcome CES-D showed a statistically significant decrease in self-reported depressiveness, with an *F* value of *F*_2,43.323_=18.94, *P*<.001. For the CES-D, 74% (20/27) of participants exhibited RCIs from pre to post assessment (deteriorations=3.7% [1/27]). Self-reported psychological distress, measured by the GHQ-12, decreased significantly, *F*_2,41.616_=12.04, *P*<.001, and RCI was found in 63% (17/27) of participants (deterioration=0% [0/27]). Estimated means, SDs, effect sizes, and RCIs of both scales are depicted in Table 3.

For applied secondary outcomes, the treatment resulted in less pronounced effects (*d*=0.38 to *d*=0.71). The AAQ-II revealed a significant change over time, *F*_2,39.710_=10.41, *P*<.001, and an effect size of *d*=0.59. A comparable pattern was found with regard to the AnTi, *F*_2,39.450_=12.68, *P*<.001, and *d*=0.72, and with regard to the PSWQ-3, *F*_2,39.447_=4.11, *P*<.001, and *d*=0.37. For further information on estimated means, SDs, and effect sizes, see Table 3.

**Table 3:** Means, SDs, effect sizes (Cohen *d*), and reliable change for primary and secondary outcomes (N=27).

Questionnaire	Estimated mean (SD)			Effect sizes (estimated mean [95% CI]), pre to post effect size	Reliable change	
	Pre	Post	Follow-up		Pre to post RCI^a^	Pre to follow-up RCI
CES-D^b^	22.44 (5.18)	13.56 (6.48)	12.19 (7.94)	1.51 (0.89 to 2.09)	74	78
GHQ-12^c^	16.07 (5.41)	9.63 (4.39)	11.94 (7.12)	1.31 (0.70 to 1.87)	63	52
AAQ-II^d^	26.15 (8.87)	20.71 (8.85)	18.63 (9.71)	0.59 (0.02 to 1.14)	—^e^	—
AnTi^f^	44.33 (10.22)	36.46 (10.45)	36.25 (11.59)	0.72 (0.14 to 1.27)	—	—
PSWQ-3^g^	7.63 (2.50)	6.67 (2.76)	6.50 (2.97)	0.37 (−0.19 to 0.91)	—	—

^a^RCI: reliable change index.

^b^CES-D: Center for Epidemiological Studies-Depression scale.

^c^GHQ-12: general health questionnaire (12-item version).

^d^AAQ-II: Acceptance and Actions Questionnaire.

^e^Not applicable.

^f^AnTi: Anxious Thoughts Inventory.

^g^PSWQ-3: Penn State Worry Questionnaire (ultra-short version).

### Maintenance of Treatment Effects

After a follow-up period of 3 months, the reduction of self-reported depression (CES-D) remained stable (*F*_1,23.556_=29.98; *P*<.001) and 78% (21/27) of participants exhibited RCI (deteriorations=7.4% [2/27]). With regard to self-reported psychological distress (GHQ-12), participants indicated significant effects from pre to follow-up, *F*_1,22.758_=4.82, *P*=.04, and RCI was found in 52% of participants (14/27) (deteriorations=11.1% [3/27]). Contrary to self-reported depressiveness, treatment effects on psychological distress regressed slightly during the follow-up period. However, these reductions failed to rise above the level of statistical significance (contrast: *t*_26_=1.39; *P*=.17). Stable treatment effects were also found for the 3 secondary outcomes: psychological flexibility (AAQ-II) *F*_1,18.867_=12.59, *P*=.002; anxious thoughts (AnTi) *F*_1,17.771_=12.04, *P*=.003; and worry (PSWQ-3) *F*_1,18.825_=4.60, *P*=.04. Further information can be obtained from Table 3.

### Client Satisfaction and System Usability

System usability of applied app and Web elements, measured by the SUS [64], unveiled an average system usability of 65.33 (SD 18.95) of 100 possible scale points. Accordingly, system usability can be classified as OK to good [65]. Participant’s service satisfaction, measured by the ZUF-8 [66], assessed an average satisfaction of mean 26.43 (*SD* 4.80) on a 32-point scale, indicating “good” client satisfaction. However, according to the weekly documentation of group sessions, group coherence in 1 group was low, and the group climate would have profited from including personality disorders (Cluster A and B) in the diagnostic procedure.

### Intervention Usage and Therapeutic Guidance

**Figure 4:**
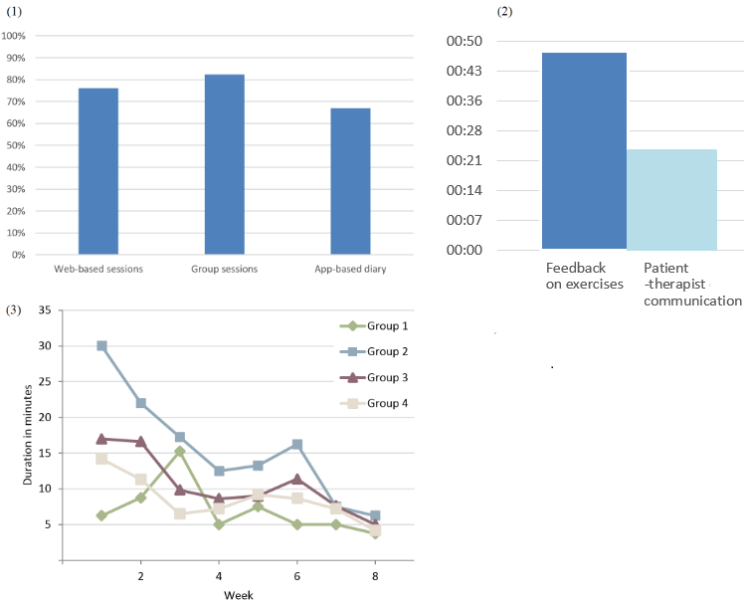
(1) Patients’ completion rates of all intervention elements. (2) Therapists’ average guidance time per patient during entire treatment; a total of 24 min was spent on personal topics, whereas 48 min was spent on feedback on specific exercises. (3) Therapists’ weekly Web-based guidance by single group.

Besides group attendance=82.4% (5.9/7 sessions), usage of digital elements was high (Figure 4): completion rate of Web-based modules 76% (5.3/7 modules), and 67% (14/21 entries) for the mobile-based diary app. However, the average number of app entries during treatment (mean 33) exhibited great variety (range 0 to 246). The average time therapists spent in the guiding of weekly Web-based modules was *mean* 10.3 min per patient, including guidance on accomplished exercises (67% or 6.9 min) and lateral patient-therapist communication (33% or 3.4 min). Thus, two-third of the total guidance was dedicated to the supervision of Web-based tasks, whereas intimate patient-to-therapist communication constituted the remaining third. There was a trend toward a reduction of guidance as the study progressed, and the single groups differed in the required guidance time.

### Therapist Interviews and Subsequent Follow-Up Survey

The therapists’ experiences with and attitudes toward bGT can be described as cautiously positive to positive. Important themes concerned the functionality and applicability of bGT and patients’ interaction with the format, as well as the general appraisal of bGT. Table 4 depicts main themes, subthemes, and frequently assigned codes of the interviews. With Cohen kappa=0.49, interrater agreement was sufficiently high. Interview results were subsequently validated by an anonymous follow-up survey, which was based on the content of the interviews (Table 5).

In the wider perspective, therapists agreed that bGT can have a positive impact on current forms of group therapy and that they had more positive attitudes toward bGT after applying this format. Perceived merits of bGT were augmented monitoring, in addition to patients’ responsiveness to given online reminders in terms of increased treatment adherence. Most therapists agreed that patients would profit from the technology-aided treatment transfer and from the repeated presentation of therapy materials (platform, app, and group sessions). Furthermore, they agreed that Web-based modules would prepare patients for subsequent group reunions. A total of 6 out of 8 therapists reported that particular patients disclosed more openly via private Web-based communication (online disinhibition effect) compared with the group meetings. Individual differences emerged in the preference of particular treatment elements. Although some therapists emphasized the added value of between-session elements, others underpinned the merits of applied in-session tools.

With regard to the potential risks of bGT, a consensus emerged that in-session media should be applied cautiously (eg, overloaded sessions) and that the intervention at times may have hampered some of the desired group dynamics (eg, too little time for discussions). In this context, the preservation of technology-free group sessions was suggested. Furthermore, 2 therapists also advocated a cautious use of Web-based reminders and prompts to prevent less interested patients from feeling overwhelmed or discouraged. During the interview, 1 therapist expressed serious concerns about data safety.

**Table 4:** Main themes, subthemes, and frequent codes of therapist interviews.

Main theme and subtheme		Frequent codes
**Advantages**		
	Patients	Content repeatable; greater learning effect; increased engagement with therapy tasks
	Therapists	Additional information through monitoring; helpful for younger therapists; guiding thread
	Interaction	Patients more open (online disinhibition); building relationship through intimate Web-based communication
**Disadvantages**		
	General	Additional effort; data security; limited management of acute crisis; predefined treatment course
	Specific	Effects on group climate and cohesion; sessions overloaded
**General evaluation**		
	Positive	Contemporary; suitable for in-patient settings; improved handling with increased routine
	Negative	Preference toward classic therapy; more training than therapy; technical issues; initial skepticism
**Web-based communication**		
	Online reminders	Require organized working style; increase compliance; unwanted effects
	Online feedback	Important feature; needs to be short in duration
**Patients’ differences**		
	Optional classic treatment path	Adaptation to patient preferences; possible side effects
	Differences in patients	Not for severe depression; amount of required guidance time; differences in media affinity; requires openness and compliance

**Table 5:** Benefits and drawbacks of blended group therapy (bGT) according to interview follow-up survey (n=8).

Statement^a^	Percentages		Mean (SD)
	Agree (rather agree)	Disagree (rather disagree)	
I am more open after experience with bGT^a^	25 (75)	0 (0)	3.25 (0.46)
I am more critical after experience with bGT^a^	0 (0)	13 (88)	1.86 (0.36)
I have serious concerns about data safety^a^	0 (25)	25 (50)	2.00 (0.76)
bGT may also be feasible for in-patient treatment^a^	13 (75)	0 (13)	3 (0.53)
Advantages of more flexible working hours because of Web-based guidance^a^	50 (38)	13 (0)	3.25 (1.03)
Computer elements^b^ should be used for in-session support^a^	50 (25)	0 (25)	3.25 (0.89)
Overuse of in-session media can hamper group dynamics^a^	50 (38)	0 (13)	3.38 (0.75)
Overuse of in-session media did hamper dynamics in my groups^a^	0 (25)	50 (25)	1.75 (0.87)
Computer elements^c^ should be used for between-session support^a^	63 (38)	0 (0)	3.63 (0.52)
Platform prepares patients optimally for group reunions^a^	38 (63)	0 (0)	3.38 (0.52)
Repeated application of therapy content fosters abilities (CE, app, and session)^a^	38 (63)	0 (0)	3.38 (0.52)
Reminders increased compliance with Web-based tasks^a^	13 (75)	0 (13)	3.00 (0.53)
bGT cannot increase treatment transfer^a^	0 (13)	38 (50)	1.75 (0.71)
Reminders did exert a lot of pressure on some patients^a^	13 (50)	0 (38)	2.75 (0.71)
Additional between-session therapist time needs to be reimbursed^a^	88 (13)	0 (0)	3.88 (0.35)
Patients shared additional private concerns over platform (online disinhibition)^a,d^	50 (34)	0 (17)	3.33 (0.82)
Between-session contact made me feel more connected with clients^a,d^	17 (83)	0 (0)	3.17 (0.41)
Between-session contact does not promote relationship with client^a,d^	0 (0)	33 (67)	1.67 (0.52)

^a^Exact wording is provided in Multimedia Appendix 2.

^b^Slides and videos.

^c^Platform, app, and monitoring.

^d^Optional questions only applied to 6 therapists.

## Discussion

### Principal Findings

This study investigated the feasibility of a mobile- and Web-supported bGT for depression, with a focus on therapists’ perception of and patients’ adherence to the novel format. High effects on self-reported depressiveness and general health, as well as beneficial effects on ACT-related secondary outcomes, were observed. Effects remained stable over a short follow-up period. Therapist interviews revealed high treatment applicability and perceived benefits concerned treatment availability and monitoring and transfer, as well as the establishment of the therapeutic relation. On an average, therapists spent 10 min per patient per week with online guidance, with decreasing guidance over the course of time and variation between individual groups. Regarding patients’ system usage, participants almost equally engaged in weekly group reunions and Web-based tasks. Usage patterns of the mobile-based diary varied to some extent.

Applied primary outcome measures indicated substantial effects on self-reported depressiveness and general health after the outpatient treatment had ended. Observed effects correspond to earlier bGT depression studies [40,41,74], to benchmarking meta-analyses on group therapy [75-77], and to recent group therapy trials in routine care [78,79]. As guideline-based group CBT usually entails 15- to 20-hour sessions [33], high treatment effects where achieved in a comparably short period of time. Although the most observed effects remained stable, self-reported general health decreased slightly but non significantly at follow-up. To further increase treatment success, different forms of online aftercare [23,24] could easily be integrated into bGT and flexible care solutions, such as discontinuous groups, booster sessions, or online groups [80,81], can be facilitated by bGT. As a related aspect, long-term effects of bGT need to be studied in future trials.

This study adds a first therapist-related perspective to the growing evidence on bGT. Retrospectively, novice therapists described the format as contemporary, featuring patient- and therapist-related, as well as interactional, advantages. They reported patients to engage intensely with the bGT tasks, leading patients to be well prepared for the next group session. Furthermore, they appreciated the format for providing flexible working hours, as well as information about the individual treatment progress. Even though not all therapists were initially fond of the novel format, personal experience increased the self-reported willingness to work with the novel approach [82,83]. As for the perceived disadvantages, therapists mentioned the additional between-session workload and some preferred a more classical format. In this context, some therapists stated that it may be difficult to attract experienced or less-interested therapists and that the treatment had a more training-like character. Furthermore, patients should not feel overwhelmed by the use of technology or the intensity of treatment (eg, reminders).

With regard to the reported improvement of therapeutic alliance, the therapist back-end system allowed personalized feedback on completed tasks (two-third of the time), as well as intimate lateral communication between therapists and clients (one-third of the time). Interviewed therapists appreciated both functionalities, and according to the therapists, patients responded to online prompts, resulting in an increased completion of outstanding therapy tasks. As a last consideration, all therapists that used software with implemented confidential communication (6 out of 8 therapists) reported that some of their patients disclosed more openly via intimate lateral communication. This phenomenon can be classified as a form of the online disinhibition effect [84]. In a previous study, the online disinhibition in bGT seemed to be fostered by the perceived intimacy between the patient and therapist in the absence of an additional audience [42].

As another important feasibility criterion, the amount of additional workload because of Web-based guidance is from particular relevance [85]. Beyond doubt, the time required by therapists depends on the implemented tasks of a given intervention. We found a moderate amount of additional workload in an intervention designed to provide close between-session guidance. Most therapists expected further reductions of required guidance time with a growing routine in conducting bGT. Therapist support is frequently associated with improved treatment adherence and lower dropout rates [15]. Here, bGT can be a reasonable alternative to existing formats, such as Web-based therapy or blended individual therapy.

Treatment flexibility is of particular interest in outpatient groups, as the scheduling of group sessions is usually restricted to evening hours on a specific weekday. On one hand, technology-induced treatment flexibility is appreciated by patients [32,42]. On the other hand, more flexible working hours may also prove to be attractive for certain therapists. Here, increases in flexibility are achieved by moving working hours toward Web-based guidance between sessions. In a double trainer setting with a group size of 8 patients, the expected Web-based guidance for 4 patients takes around 45 min per therapist and week. In this regard, surveyed therapists uniformly emphasized the relevance of reimbursement for Web-based guidance time. Even though this additional workload can easily be compensated by shortening the overall treatment duration [28], such shortenings should be carried out carefully and in accordance with patient needs [86] (eg, time to establish trust in the group).

bGT takes a special position in the field of internet interventions. First, bGT can be a cost-efficient treatment option situated between guided Web-based interventions and blended individual therapy (Figure 1). Compared with Web-based interventions, bGT preserves real-world contact at slightly higher costs. When compared with individual therapy, however, bGT can lead to similar cost savings as known from classical group therapy. Second, group phenomena could be harnessed to support therapist efforts to promote compliance with Web-based tasks [42,87]. Compared with Web-based interventions that sometimes suffer from low adherence rates [15,88], patients engaged to a wide extent in the featured Web-based tasks, as results indicate comparable adherence to group sessions and technology-based elements. Finally, bGT blurs distinctions between individual and group therapy, as it brings a high degree of individualized care to the group format. For example, it opens new ways for intimate patient-to-therapist communication, and it routinely provides therapists with individual information on treatment progress or potential problems [74].

With regard to potential disadvantages of bGT, therapists mentioned that certain participants may feel overwhelmed by the close monitoring of between-session activities or by the number of set reminders. For this reason, the intensity of monitoring and Web-based activities should be adaptable to patient needs. As a second aspect, 2 therapists expressed concerns about data safety. These concerns should be treated with high priority to prevent therapists from being deterred. Third, extensive in-session media use was described as a risk factor, potentially dampening desired group dynamics. Although observable incidences were reported less frequently (Table 5), bGT interventions can profit from a cautious implementation of in-session technology. Fourth, some therapists stated that it may be more difficult to attract experienced or less interested therapists. Thus, incentives seem important to make bGT a workable approach (eg, reimbursement of Web-based guidance time, flexibility of working hours, and balance of work tasks). As a last aspect, 1 therapist mentioned the limited management of acute crisis, which theoretically may be induced by Web-based elements or between-session tasks. Here, technology can provide new ways of emergency management too, for example, by the installation of an emergency button, as seen in a blended app-supported problem-solving treatment for patients with intentional self-harm [89].

This study has several noteworthy strengths and limitations. First, this study adds a first therapist-related perspective to previous findings on bGT [37-42]. Second, it applies a multimodal research strategy (eg, triangulation of quantitative and qualitative methods and implementation of log data) to investigate feasibility in a more holistic way. Third, in accordance with recommendations on the documented use of technology [90], this study provides detailed and objectively measured information on Web-based and mobile app completion rates. Fourth, compared with previous bGT depression studies [40,41] and studies on Web-based interventions [91,92], the current sample composition is more balanced with regard to gender and the level of education. Finally, the study reports deterioration rates and possible risks associated with the novel format.

Among its most important limitations, this study was designed and powered to investigate the feasibility of bGT for depression. The study design, therefore, does not allow any conclusions about technology-induced increases in efficiency or effectiveness. Together with blended individual therapy trials [29,30], future research will have to determine the merits of bGT in terms of augmented treatment effects. Second, many different constellations of blended therapy exist and heterogeneity within the field is high [23]. At hand, findings primarily represent the more integrated forms of blended therapy, whereas less-integrated forms (eg, adjunct Web-based programs) may differ in the therapist’s guidance, the flexibility of treatment, or the intensity of treatment. In this context, bGT concepts for group psychotherapy (>15 to 20 sessions), as well as blends of internet interventions with telegroup therapy [93,94], and discontinuous groups should be developed. Third, even though conducted in an outpatient clinic, the study setting restricts generalizability, as groups were held at an affiliated university center for psychotherapy and counseling, and the sample was self-selected. Therefore, it is probable that clients were more interested in this kind of treatment. Furthermore, the treatment was carried out by novice therapists. Although some study aspects appear less prone to introducing bias (ie, Web-based guidance time or log files), it is likely that novice therapists are more adaptable to innovations. More ample evaluations of therapist views exist in neighboring fields, such as individual blended therapy, tele therapy, and Web-based therapy [16,31,93,94].

### Conclusions

This study adds a first therapist perspective to previous research on bGT. Feasibility was supported within a university outpatient setting, treating a demographically balanced sample with a short but intense ACT-based group intervention. Even though the intervention entailed a variety of Web- and app-based elements, the amount of online guidance was manageable, and guidance resulted in more flexible working hours. The Web-based platform was appreciated for the implementation of between-session monitoring and the establishment of therapeutic alliance. According to therapists, compliance with CBT tasks can be fostered by prompts via the Web-based platform, resulting in high adherence rates. Potential negative effects of blending should be regarded in the design and implementation of bGT interventions.

## Appendix

Multimedia Appendix 1 Interview guide.

Multimedia Appendix 2 Subsequent questionnaire based on previous therapist-interviews.

## Abbreviations

AAQ-II: acceptance and action questionnaire-II
ACT: Acceptance and Commitment Therapy
AnTi: Anxious Thoughts Inventory
BA: Behavioral Activation
bGT: Blended Group Therapy
CBT: Cognitive Behavioral Therapy
CES-D: Center of Epidemiologic Studies Depression
DIPS: Diagnostic Interview for Psychological Disorders
GAD: Generalized Anxiety Disorder
GHQ: General Health Questionnaire
ITT: intention-to-treat
PSWQ: Penn State Worry Questionnaire
RCI: Reliable Change Index
SUS: System Usability Scale
ZUF-8: Fragebogen zur Patientenzufriedenheit
