# Supplementary material for: Effects, Adherence, and Therapists’ Perceptions of Web- and Mobile-Supported Group Therapy for Depression: Mixed-Methods Study
Source: J Med Internet Res. 2019 May 6;21(5):e11860. doi: 10.2196/11860 (PMC6533044; doi:10.2196/11860)
Supplement: Multimedia Appendix 1 [file jmir_v21i5e11860_app1.pdf]

Appendix 1

Interview guide

What do you associate with this therapy format (bGT)? What comes to your mind?

What is your general evaluation of the blended format?

How did patients use and perceive the computer elements?
- In-session media?
- Between-session media (platform, app)?
- For whom is it suitable and for whom not?

What are strengths and weaknesses of the bGT approach?

What do you think about the monitoring of homework activity?

How does the blended format influence your general perception of a given individuals' therapy progress?

How did you interact with patients via the platform? Were there any important moments?

How does bGT effect the application of evidence-based treatments?

What should therapists keep in mind when applying blended group therapy?

How would you describe the applicability and the weekly workload of bGT from a therapist's perspective?

What problems did you experience with the blended format?

What do you think about the application of bGT in routine care?
