# Supplementary material for: Effects, Adherence, and Therapists’ Perceptions of Web- and Mobile-Supported Group Therapy for Depression: Mixed-Methods Study
Source: J Med Internet Res. 2019 May 6;21(5):e11860. doi: 10.2196/11860 (PMC6533044; doi:10.2196/11860)
Supplement: Multimedia Appendix 2 [file jmir_v21i5e11860_app2.pdf]

Appendix 2

Subsequent questionnaire based on previous therapist-interviews

1. Computer-aided elements (platform, app, monitoring) should be applied between sessions.
2. Computer-aided elements (PowerPoint slides, videos, etc.) should be applied during sessions.
3. Providing two intervention paths: If possible, blended group therapy should be designed in a way that less interested persons can chose between a classical participation (notebook and workbook) and a technology-aided participation. [a]
4. What is the optimal proportion of in-session media support in a 90 minutes session (in minutes, slider)? [a]
5. How many sessions should be implemented without in-session media support (in percent, slider)? [a]
6. Too much in-session media can affect group dynamics negatively.
7. In-session media did effect my groups negatively.
8. Some patients shared personal issues via the platform that they did NOT address in the group (online disinhibition).
9. The online feedback made me feel more related to the participants between group sessions.
10. Between-session contact did NOT increase the therapeutic relationship with participants.
11. The online reminder did increase compliance with online exercises.
12. The online reminders caused too much pressure for certain patients (excessive demand, demotivation).
13. The online platform prepared participants optimally for the upcoming group session.
14. The repeated processing of content (online, then group, then app) facilitates learning of therapeutically relevant contents and abilities.
15. The blended format can NOT improve transfer into daily life.
16. For me, a decline in usage of online elements relates to habituation. (Initial curiosity and interest fade to habituation and routine – a natural process) [a]
17. For me, a decline in usage relates to technical and design aspects. (The used media should be better implemented, so one can expect less decline) [a]
18. I connect a decline in usage with the following: (open format) [a]
19. What is the maximum group size, if detailed between-session online feedback is required in a bGT intervention (double trainer format)? [a]
20. What is the maximum group size, if detailed between-session online feedback is NOT required / is mainly automatized? [a]
21. I am more open-minded towards blended therapy than I was at the beginning of the training (When I didn't know the format).
22. I am more critical about the blended format than I was at the beginning of the training (When I didn't know the format).
23. bGT could be suitable for the inpatient setting.
24. The extra effort (e.g. between-session guidance) needs to be reimbursed. (Time must be considered as working hours).
25. The additional work load can be compensated by a shortening of overall treatment duration.
26. Between-session guidance leads to more flexibility of working hours. This is an advantage, because ambulant group therapy is scheduled during evening hours.
27. I have serious concerns regarding data security in bGT.
28. What are the three most important advantages of bGT? (open format) [a]
29. What are the three most important disadvantages of bGT? (open format) [a]

Note: a = will be reported in a further publication on bGT design
